# Key motor and non-motor features in early dementia with Lewy bodies

**DOI:** 10.3389/fneur.2025.1555175

**Published:** 2025-03-13

**Authors:** Anna Planas-Ballvé, José Rios, Lourdes Ispierto, Mireia Gea, Laia Grau, Marta Jiménez, Cynthia Cáceres, Sílvia Martínez, Katrin Beyer, Ramiro Álvarez, Pau Pastor, Dolores Vilas

**Affiliations:** ^1^Movement Disorders Unit, Neurology Department, Complex Hospitalari Moisès Broggi, Barcelona, Spain; ^2^Movement Disorders Unit, Neurology Department, Hospital Universitari Germans Trias i Pujol, Barcelona, Spain; ^3^Department of Clinical Pharmacology, Hospital Clinic, IDIBAPS, Biostatistics Unit, School of Medicine, Universitat Autònoma de Barcelona, Barcelona, Spain; ^4^Epilepsy Unit, Neurology Department, Hospital Universitari Germans Trias i Pujol, Badalona, Spain; ^5^Neuropsychology Unit, Neurology Department, Hospital Universitari Germans Trias i Pujol, Badalona, Spain; ^6^Department of Pathology, Hospital Universitari Germans Trias i Pujol, Badalona, Spain; ^7^Department of Medicine, Universitat Autònoma de Barcelona, Barcelona, Spain

**Keywords:** dementia with Lewy bodies, motor symptoms, non-motor symptoms, neurodegenerative diseases, differential diagnosis

## Abstract

**Objective:**

The objective of our study was to characterize early-stage dementia with Lewy bodies (DLB) focusing on motor and non-motor symptoms.

**Methods:**

This cross-sectional study prospectively included newly diagnosed DLB patients within 3 years of cognitive symptom onset. Comparisons were made with individuals with Parkinson’s disease (PD), Alzheimer’s disease (AD), and controls. Demographic and clinical data were collected, and motor and non-motor symptoms were assessed using structured interviews and validated scales and questionnaires.

**Results:**

A total of 107 participants were included (23 DLB, 27 PD, 26 AD, and 31 controls). DLB patients (median age 75 years, median disease duration since diagnosis 2 months) commonly reported motor symptoms, including gait disturbances (91.3%), tremor (73.9%), and bradykinesia (87%), with tremor being predominantly unilateral (76.5%) and action-type (52.9%). The most frequent motor subtype was akinetic-rigid (52.2%). Motor symptoms were similar to PD, except for more frequent falls (34.8% vs. 11.1%, *p* = 0.044) and gait disturbances in DLB patients (91.3% vs. 63%, *p* = 0.019). Non-motor symptoms, particularly visual hallucinations and neuropsychiatric symptoms were more prevalent in DLB than in PD, while sleep and autonomic symptoms were similar. An abnormal orthostatic test was more frequent in DLB than in PD (45.5% vs. 11.5%, *p* < 0.008). Compared to AD, all non-motor symptoms were significantly more frequent in DLB. Finally, DLB patients had lower functional independence and quality of life than both PD and AD (*p* < 0.0001).

**Conclusion:**

Early-stage DLB closely resembles PD in motor symptoms but has more neuropsychiatric non-motor symptoms compared to PD and overall non-motor symptoms than AD.

## Introduction

Dementia with Lewy bodies (DLB) is a neurodegenerative disorder that is increasingly recognized as a prevalent cause of dementia, accounting for approximately 5% of all dementia cases (1, 2). With the progressive aging of the global population, it is expected that prevalence and incidence of DLB will increase in the next decades (3). The economic and social burden associated with DLB is substantial, with higher hospitalization rates and greater healthcare utilization compared to other types of dementia (4, 5). Therefore, a better understanding of the disease, especially in its earlier stages, is crucial for improving early and accurate diagnosis.

DLB is characterized by cognitive decline associated with a variable combination of visual hallucinations, fluctuations in cognition, parkinsonism and REM sleep behavior disorder (RBD). The prodromal phase of DLB, includes mild cognitive impairment (MCI), delirium-onset, and psychiatric-onset presentations. The MCI-DLB onset is the most studied and is usually characterized by deficits in attention, executive function, and visuospatial abilities, with relatively milder memory impairment, and is often accompanied by symptoms such as RBD, autonomic dysfunction, visual hallucinations and subtle motor signs (6). The current diagnostic criteria for DLB and its prodromal MCI stage rely on clinical features and proposed biomarkers that classify patients as either possible or probable DLB or prodromal DLB (7, 8). However, the absence of a disease-specific biomarker for DLB complicates accurate diagnosis, especially given the overlap of clinical features with other neurodegenerative diseases such as Alzheimer’s disease (AD) and Parkinson’s disease (PD). Nonetheless, promising biomarkers for detecting misfolded α-synuclein have emerged through the seed amplification assays (SAA), including real-time quaking-induced conversion (RT-QuIC) and protein misfolding cyclic amplification (PMCA), highlighting SAA’s potential as a reliable DLB biomarker (9).

Previous studies have explored the prodromal manifestations of DLB, particularly in the context of MCI, offering valuable insights into its early symptoms and progression (6, 10–14). These studies suggest that motor symptoms, such as parkinsonism, can be present in the early stages of DLB, although their prevalence varies widely, ranging from 15 to 70%. Nevertheless, the majority of these previous studies have focused on patients from memory clinics, leading to a limited description of motor symptomatology in the context of initial symptoms. In addition, non-motor symptoms such as RBD, visual hallucinations and autonomic dysfunction are commonly observed and often precede cognitive decline (15).

Our study aims to provide a more comprehensive clinical characterization of the early stages of DLB, with a particular focus on motor symptoms and non-motor manifestations other than the cognitive decline.

## Methods

### Study design and patient selection

This cross-sectional study was conducted from January 2021 to January 2023 at the Neurodegenerative Diseases and Movement Disorders Unit of Hospital Universitari Germans Trias i Pujol. Participants were prospectively recruited from the outpatient clinic. We included newly diagnosed patients who fulfilled the current clinical criteria for probable DLB (7). At inclusion, all patients were specifically asked about the onset of motor and cognitive symptoms, and only those reporting symptom onset within the last 3 years were considered eligible. Additionally, patients had to present a Global Deterioration Scale (GDS) score of 4 or less (16). For comparative analysis, we also recruited patients with PD and AD, who fulfilled the Movement Disorders Society (MDS) criteria for PD (17) and the National Institute on Aging and Alzheimer’s Association (NIA-AA) criteria for AD (18), respectively. In both groups of patients, the time from onset of motor or cognitive symptoms, respectively, was less than 3 years, and the score in the GDS up to four. A control group was also included, consisting of non-blood relatives of the study participants who had no known neurological diseases. This study was approved by the Ethics Committee of Hospital Germans Trias i Pujol (PI-18-114), and all participants gave their written consent to participate in the study and use their clinical data for research purposes.

### Clinical assessment

Demographic, clinical data and current medications were collected from all participants. The date of diagnosis was recorded and the date of the disease onset was considered as the date of the first cognitive symptom reported. Diagnostic delay was calculated using the onset of motor or cognitive symptoms and the date of diagnosis, excluding 2 PD patients with essential tremor lasting over 20 years before diagnosis. For DLB patients, we also reviewed their medical history to determine the reasons for their referral to the neurologist (motor, cognitive, psychiatric or sleep-related complaints).

Motor features were explored by means of a structured interview performed by a movement disorders specialist, documenting whether participants presented tremor (recording the initially affected side, the part of the body firstly involved and the circumstances under which it was triggered), bradykinesia, falls, gait disturbances, hypophonia and micrographia. An evaluation of the presence of parkinsonian signs was done, by an experienced Movement Disorders specialist (APV), by means of the MDS Unified Parkinson’s Disease Rating Scale (MDS-UPDRS) Parts II and III (19) and the Hoehn and Yahr scale (H&Y scale) (20). The severity of tremor and bradykinesia was calculated as the sum of the corresponding MDS-UPDRS III items. Additionally, DLB and PD patients were classified into tremor-dominant (TD), postural-instability and gait disturbance (PIGD), and indeterminate subtypes according to predefined ratio thresholds using the MDS-UPDRS (21). We also reviewed whether Dopamine transporter (DaT) scans had been performed as part of routine clinical practice and their corresponding results.

The presence of non-motor symptoms was determined according to the PD Non-Motor Symptoms Questionnaire (NMSQuest) (22) and the MDS-UPDRS Part I (19). The diagnosis of clinically probable RBD was made using the RBD Single-Question Screen (RBD1Q) (23), and excessive daytime somnolence was assessed with the Epworth Sleepiness Scale (ESS) (24). Autonomic dysfunction was evaluated using the Scales for Outcomes in PD-Autonomic (SCOPA-AUT) (25) and an orthostatic blood pressure test conducted in supine position and after standing for 1 and 3 min. The Neuropsychiatric Inventory (NPI) (26) and the Hospital anxiety and depression scale (HADS) (27) were performed to assess psychiatric and mood disturbances, while the 4-item Mayo Fluctuations Scale was used to evaluate cognitive fluctuations, defined as present if the scale score was ≥3 (28). The presence of major and minor visual hallucinations (including passage and presence hallucinations) was also recorded through a structured interview, specifically inquiring about their occurrence in the past 3 months. The cognitive screening tools Minimental State Examination (MMSE) (29) and Montreal Cognitive Assessment (MOCA) (30), as well as the GDS were also used. The EuroQol-5 Dimension (EQ-5D) (31) and the Schwab & England scale (S&E scale) (32) were applied for a general view of quality of life and functional independence.

### Statistical analysis

Descriptive demographical and clinical data are presented as median values with interquartile ranges (IQR; 25th and 75th percentiles) or as number and percentages. For group comparisons of qualitative variables, we used the Chi-square test. For quantitative variables, the Kruskal-Wallis test was applied for comparisons involving more than two groups, and the Mann–Whitney U test was used for comparisons between two groups. All statistical analyses were performed using SPSS Version 26 (IBM Corp. Armonk, NY, USA). Differences were considered statistically significant at a two-sided type I error rate of 0.05.

## Results

### Demographics and baseline characteristics

One hundred and seven participants were included in the study: 23 patients with DLB, 27 PD, 26 AD, and 31 controls. The demographic and clinical characteristics of the participants are summarized in Table 1. The median age of DLB patients was 75 (72–77) years, similarly to the other study groups (*p* = 0.079). Seventeen (73.91%) DLB patients were men, a percentage similar to PD patients (62.96%), but higher than AD and control groups (38.46 and 38.71%, respectively; *p* = 0.02). Patients were included in the study after 2 (0–4) months following diagnosis, and 24 (15–36) months since the onset of cognitive symptoms. The median diagnostic delay was 2.6 (1.9–3.0) years in DLB, 1.9 (1.3–3.2) years in PD, and 3.3 (2.3–4.6) years in AD.

**Table 1:** Demographic and clinical data of study participants.

	DLB (*n* = 23)	PD (*n* = 27)	AD (*n* = 26)	Controls (*n* = 31)	*p*-value	*p*-value DLB vs PD	*p*-value DLB vs AD
Age (years)	75 (72–77)	70 (64–75)	73.5 (68–76)	72 (63–77)	0.079		
Sex (male)	17 (73.91)	17 (62.96)	10 (38.46)	12 (38.71)	0.02	0.546	0.021
Arterial hypertension	16 (69.6)	15 (55.6)	12 (46.2)	9 (29)	0.025	0.309	0.097
Diabetes mellitus type 2	5 (21.7)	6 (22.2)	3 (11.5)	4 (12.9)	0.611		
Dyslipidemia	15 (65.2)	16 (59.3)	12 (46.2)	17 (54.8)	0.583		
Time from diagnosis to study evaluation	2 months (0–4)	10 months (0–26)	2 months (0–5)		0.029	0.027	0.079
Motor symptom onset to evaluation time	24 months (12–36)	24.5 months (12–55)				0.283	
Cognitive complaints	23 (100)	10 (37)	25 (96.15)		<0.001	<0.001	0.342
Cognitive symptom onset to evaluation time	24 months (15–36)	24 months (12–60)	36 months (24–48)		0.065		
Diagnostic delay	2.6 years (1.9–3)	1.9 years (1.3–3.2)	3.3 years (2.3–4.6)		0.011	0.196	0.040
Treatment							
Levodopa	14 (60.9)	21 (77.8)					
LEDD	300 mg (0–450)	300 mg (100–300)				0.508	
Dopamine agonist treatment	0	10 (37)				<0.001	
MAO-B inhibitors treatment	0	5 (18.5)				0.01	
AChEIs	12 (52.2)	0	22 (88)	0			0.006
Antidepressant use	9 (39.1)	5 (18.5)	12 (48)	6 (19.4)	0.044	0.106	0.109

DLB, Dementia with Lewy bodies; PD, Parkinson’s disease; AD, Alzheimer’s disease; LEDD, levodopa equivalent daily dose; MAO-B inhibitors, Monoamine Oxidase Type B inhibitors; AChEIs, Acetylcholinesterase inhibitors.

Quantitative data are expressed as median and interquartile range (IQR). Qualitative variables are expressed as numbers and percentages.

At the time of the study visit, 12 (60.9%) DLB patients were taking levodopa, with a daily levodopa equivalent dose (LEDD) of 300 mg (0–450). There were no statistically significant differences between DLB and PD patients in the percentage of those taking levodopa (77.8% in PD, *p* = 0.193) or in the LEDD (*p* = 0.508).

The reasons for referral to the neurologist in the DLB group were heterogeneous: 34.8% were primarily due to cognitive complaints, 21.7% due to motor issues, 13% due to psychiatric symptoms, and 30.4% related to sleep disturbances.

### Motor symptoms

**Figure 1:**
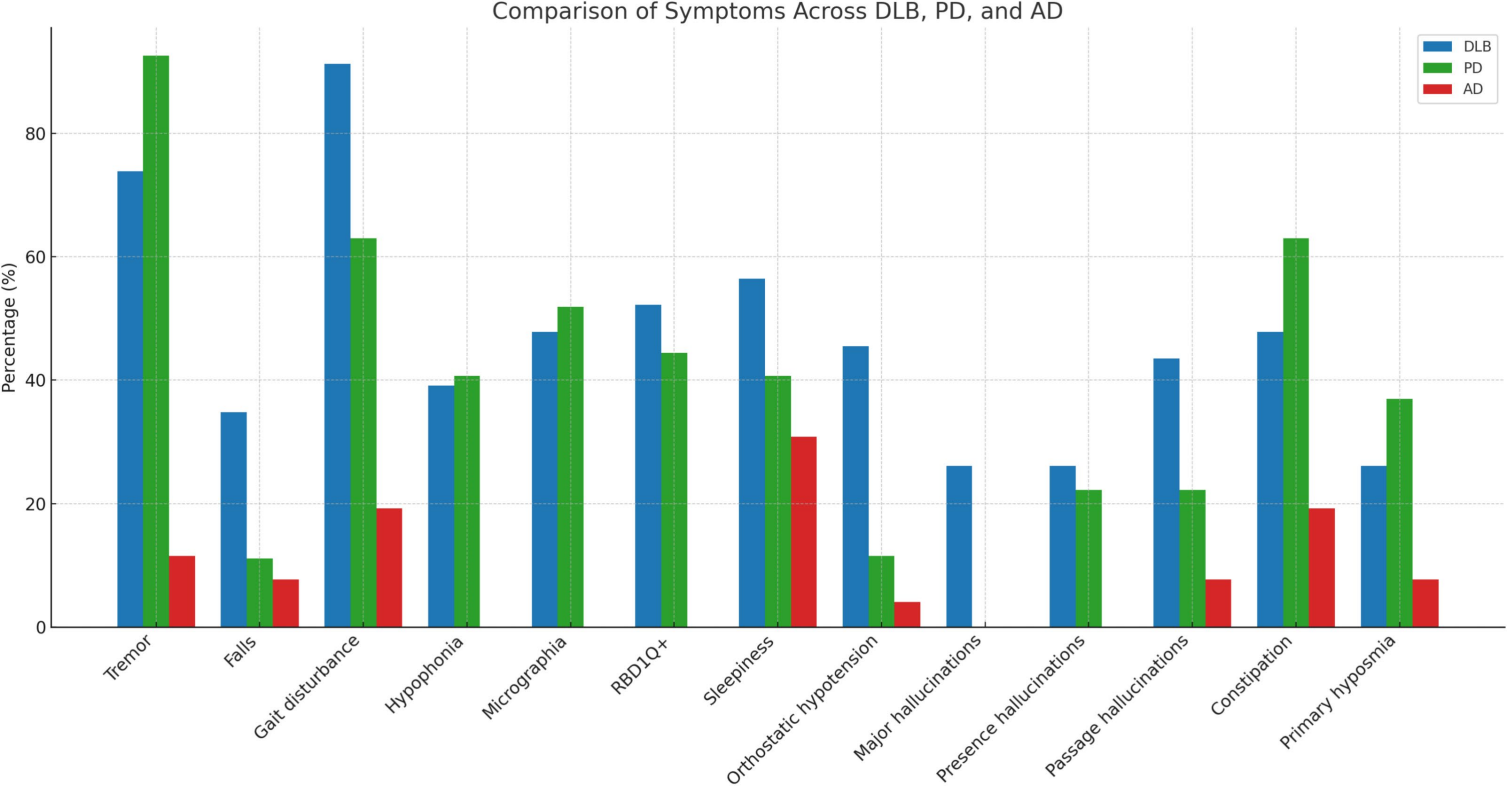
Comparison of motor and non-motor symptom frequencies across DLB, PD, and AD groups.

The majority of DLB patients reported, during the structured interview, having experienced motor symptoms. Twenty-one patients (91.3%) reported gait disturbances, 17 (73.9%) tremor, and 20 (87%) bradykinesia (Figure 1). Among patients with tremor, 9 out of 17 (52.9%) described it as an action tremor, 4 (23.5%) as a rest tremor, and 4 (23.5%) as a mixed tremor. Tremor was predominantly unilateral in 13 cases (76.5%), affecting the right side in 9 patients (52.9%). Other motor symptoms, such as micrographia or hypophonia were less frequent (47.8 and 39.1%, respectively). Overall, motor symptoms, as reported by the patients, appeared approximately at the same time as cognitive symptoms, with both having a median duration of 24 months prior to the time of evaluation.

The percentage of motor symptoms reported by the patients was similar between the DLB and PD groups, as summarized in Table 2. Only falls and gait disturbances were significantly more prevalent in the DLB group compared to PD patients (34.8% vs. 11.1%, *p* = 0.044; and 91.3% vs. 63%, *p* = 0.019, respectively). In contrast, AD patients reported motor symptoms much less frequently (Table 2).

**Table 2:** Motor symptoms of DLB patients, compared to AD and PD patients.

	DLB (*n* = 23)	PD (*n* = 27)	AD (*n* = 26)	Controls (*n* = 31)	*p*-value	*p*-value DLB vs PD
Structured interview						
**Tremor**	17 (73.9)	25 (92.6)	3 (11.5)	2 (6.5)	<0.001	0.073
*Time since tremor onset*	24 months (12–36)	24.5 months (12–55)	12 months (12–12)	66 months (12–)	0.357	0.283
*Tremor laterality*					0.228	0.339
Right	9 (52.9)	11 (44)	1 (33.3)	1 (50)		
Left	4 (23.5)	11 (44)	0	0		
Bilateral symmetric	4 (23.5)	3 (12)	2 (66.7)	1 (50)		
*Activation conditions of tremor*					0.045	0.083
Rest	4 (23.5)	11 (44)	0	0		
Action	9 (52.9)	5 (20)	3 (100)	2 (100)		
Mixed	4 (23.5)	9 (36)	0	0		
**Bradykinesia**	20 (87)	24 (88.9)	3 (11.5)	1 (3.2)	<0.001	0.834
*Time since bradykinesia onset*	24 months (12–36)	12.5 months (12–25)	24 months (12–)		0.692	0.425
*Bradykinesia laterality*					0.169	0.141
Right	5 (25)	6 (25)	0	0		
Left	4 (20)	11 (45.8)	0	0		
Bilateral symmetric	11 (55)	7 (29.2)	3 (100)	1 (100)		
**Falls**	8 (34.8)	3 (11.1)	2 (7.7)	1 (3.2)	0.005	0.044
**Gait disturbance**	21 (91.3)	17 (63)	5 (19.2)	4 (12.9)	<0.001	0.019
*Time since gait disturbance onset*	23 months (12–24)	18 months (11–36)	12 months (12–47)	58 months (44–)	0.188	0.936
**Hypophonia**	9 (39.1)	11 (40.7)	0	0	0.908	
**Micrographia**	11 (47.8)	14 (51.9)	0	0	0.777	
Validated motor scales						
MDS UPDRS II	9 (3–14)	6 (3–8)	0 (0–1)	0 (0–0)	<0.001	0.023
MDS UPDRS III	19 (13–33)	20 (13–24)	2 (1–6.25)	0 (0–2)	<0.001	0.930
Severity of tremor	1 (0–4)	3 (1–7)				0.062
Severity of bradykinesia	8 (3–18)	9 (5–12)				0.822
H&Y scale						0.007
0	0	0				
1	0	6 (22.2)				
2	19 (82.6)	21 (77.8)				
3	4 (17.4)	0				
Motor subgroups						0.018
TD	6 (26.1)	17 (63)				
PIGD	15 (65.2)	10 (37)				
Indeterminate	2 (8.7)	0				

DLB, Dementia with Lewy bodies; PD, Parkinson’s disease; AD, Alzheimer’s disease; MDS-UPDRS, Movement Disorders Society Unified Parkinson’s Disease Rating Scale; H&Y scale, Hoehn and Yahr scale; TD, tremor-dominant; PIGD, postural-instability and gait disturbance.

Quantitative data are expressed as median and interquartile range (IQR). Qualitative variables are expressed as numbers and percentages.

MDS-UPDRS part III scores were similar between DLB and PD patients (19 (13–33) and 20 (13–24), respectively; *p* = 0.930), as well as the median tremor and bradykinesia severity (*p* = 0.062 and *p* = 0.822, respectively). However MDS-UPDRS part II scores, assessing motor experiences of daily living, were significantly higher in DLB patients than in PD patients (9 (3–14) vs. 6 (3–8); *p* = 0.023) (Table 2). The majority of both DLB and PD patients had H&Y scale stage of 2 (82.6 and 77.8%, respectively). After the analysis of motor subgroup distribution, we observed that 15 (65.2%) of DLB patients were classified as PIGD, 6 (26.1%) as TD, and 2 (9.8%) as indeterminate, while PD patients were predominantly classified as TD (63%) (*p* = 0.018) (Table 2). DaT scans were performed as part of routine clinical practice in 19 (82.6%) DLB patients and 6 (22.2%) PD patients. Abnormal results were observed in 18 (94.7%) of the DLB patients and all 6 (100%) PD patients.

### Non-motor symptoms

DLB patients showed higher scores in global non-motor symptoms questionnaires, such as the NMSQuest and the MDS-UPDRS Part I, compared to AD and PD patients. Data about non-motor symptoms between patients is shown in Table 3.

**Table 3:** Nonmotor symptoms of DLB patients, compared to AD and PD patients.

	DLB (*n* = 23)	PD (*n* = 27)	AD (*n* = 26)	Controls (*n* = 31)	*p* valor	*p* value DLB vs PD	*p* value DLB vs AD
Cognitive fluctuations	5 (21.7)	0	2 (7.7)	0	0.005	0.011	0.161
MDS-UPDRS part I	14 (10–17)	8 (4–11)	6.5 (3.75–10.25)	4 (1–6)	<0.001	0.002	
NMSQuest	11 (8–14)	7 (4–10)	4.5 (3–7)	4 (2–6)	<0.001	0.007	<0.001
RBD1Q +	12 (52.2)	12 (44.4)	0	1 (3.2)	<0.001	0.586	
Sleepiness (ESS ≥ 7)	13 (56.5)	11 (40.7)	8 (30.8)	5 (16.1)	0.017	0.266	0.069
SCOPA AUT Total	16 (14–23)	17 (13–20)	9 (6.75–13)	12 (7–18)	0.001	0.654	<0.001
SCOPA Gastrointestinal	4 (2–7)	4 (2–5)	1.5 (1–3)	2 (0–4)	0.001	0.266	0.001
SCOPA Urinary	7 (5–11)	7 (3–9)	3.5 (2–6)	5 (3–7)	0.016	0.664	0.005
SCOPA Cardiovascular	1 (0–3)	0 (0–1)	0 (0–1)	0 (0–1)	0.089	0.045	0.040
SCOPA Thermoregulatory	0 (0–2)	1 (0–2)	0 (0–1.25)	0 (0–3)	0.436	0.779	0.280
SCOPA Pupillomotor	0 (0–1)	1 (0–1)	0 (0–2)	0 (0–1)	0.630	0.339	0.904
SCOPA Sexual	4 (3–6)	3.5 (0–6)	4 (2–5)	4 (0–4)	0.335	0.207	0.312
Orthostatic hypotension test	10 (45.5) (22 evaluated)	3 (11.5) (26 evaluated)	1 (4) (25 evaluated)	1 (3.4) (29 evaluated)	<0.001	0.008	
Major visual hallucinations	6 (26.1)	0	0	0	<0.001	0.005	
Presence hallucinations	6 (26.1)	6 (22.2)	0	0	0.001	0.750	
Passage hallucinations	10 (43.5)	6 (22.2)	2 (7.7)	0	<0.001	0.108	
Constipation	11 (47.8)	17 (63)	5 (19.2)	5 (16.1)	<0.001	0.283	0.033
NPI total	10 (3–19)	4 (1–6)	3 (1–9)	3 (1–5)	0.001	0.004	0.004
NPI distress	8 (3–9)	2 (1–4)	2 (1–5.5)	1 (1–2)	<0.001	<0.001	0.002
HADA total	4 (3–8)	4 (3–6)	3 (1–7.25)	4.5 (2–8)	0.260	0.366	0.081
HADD total	8 (6–10)	5 (3–8)	4 (2–5)	3 (1.75–7)	<0.001	0.005	<0.001
Primary hyposmia	6 (26.1)	10 (37)	2 (7.7)	4 (12.9)	0.034	0.408	
S&E scale	80 (60–90)	90 (80–100)	100 (97.5–100)	100 (100–100)	<0.001	0.004	<0.001
EQ-5D	60 (53–70)	76 (70–79)	100 (79–100)	100 (79–100)	<0.001	<0.001	<0.001
MEC-35	30 (25.25–31.75)	34 (31.5–35)	27.50 (25–30.75)	34 (32–35)	<0.001	<0.001	0.374
MoCA	18 (14.5–22)	25 (23–27)	16 (12.5–19.75)	26 (24.5–28)	<0.001	<0.001	0.247
GDS					<0.001	<0.001	0.492
2	0	21 (77.8)	0				
3	3 (13)	6 (22.2)	1 (3.8)				
4	14 (60.9)	0	18 (69.2)				

DLB, Dementia with Lewy bodies; PD, Parkinson’s disease; AD, Alzheimer’s disease; MDS-UPDRS, Movement Disorders Society Unified Parkinson’s Disease Rating Scale; NMSQuest, Non-motor symptoms questionnaire; RBD1Q+, rapid eye movement sleep behavior disorder screening questionnaire; ESS, Epworth sleepiness scale; SCOPA AUT, Scales for outcomes in PD Autonomic; NPI, Neuropsychiatric Inventory; HADA, Hospital anxiety and depression scale – anxiety score; HADD, Hospital anxiety and depression scale – depression score; S&E scale, Schwab and England Activities of Daily Living scale; EQ-5D, EuroQol 5-Dimension Health Questionnaire; MEC-35, Spanish version of the Mini-Mental State Examination; MoCa, Montreal Cognitive Assessment. GDS, Global deterioration scale.

Quantitative data are expressed as median and interquartile range (IQR). Qualitative variables are expressed as numbers and percentages, except for the EQ-5D scale, where results are presented as mean in percentage (%) and standard deviation.

Sleep disturbances were prominent in DLB patients, with 12 (52.2%) experiencing RBD, assessed by means of the RBD1Q, and 13 (56.5%) daytime somnolence, measured by the ESS. No significant differences were found between DLB and PD in either RBD or daytime somnolence. None of the AD patients reported RBD and no significant differences were found between DLB and AD in daytime somnolence (Table 3).

SCOPA-AUT scores were similar between DLB and PD patients (16 (14–23) vs. 17 (13–20), *p* = 0.654), and higher than in AD patients (9 (6.75–13), *p* < 0.001). The most affected domains in DLB patients were urinary, gastrointestinal, and sexual, which was similar to PD patients. The bedside orthostatic test was abnormal in a higher percentage of DLB patients compared to PD and AD (45.5% vs. 11.5% vs. 4%, respectively; *p* < 0.001).

Regarding neuropsychiatric symptoms, presence and passage hallucinations were reported at higher rates in DLB patients (26.1 and 43.5%, respectively) compared to PD patients (22.2% for both) and AD patients (7.7% for passage hallucinations). Major visual hallucinations in the last 3 months were exclusively reported by DLB patients (26.1%) and were absent in PD and AD groups. Additionally, DLB patients had significantly higher scores on the total NPI compared to PD and AD (10 vs. 4 vs. 3, *p* = 0.004). Similarly, caregiver distress scores on the NPI were higher in DLB patients compared to PD and AD. Regarding depressive symptoms, the HADD scale showed significantly higher scores in DLB patients compared to PD and AD (*p* = 0.005 and *p* < 0.001). Five (21.7%) DLB patients, 2 (7.7%) AD patients and none of the PD patients had cognitive fluctuations (Table 3).

Constipation, as assessed by the NMSQuest scale, was more frequently reported in DLB and PD patients compared to AD and controls (11 (47.8%) vs. 17 (63%) vs. 5 (19.2%) vs. 5 (16.1%)). Significant differences were found between DLB and AD (*p* = 0.033), whereas no significant differences were observed between DLB and PD (*p* = 0.283) (Table 3).

Finally, DLB patients reported lower functional independence according to S&E scale scores and lower quality of life with a higher score on the mean EQ-5D, compared to PD and AD (*p* < 0.001).

## Discussion

In this study, we aimed to provide a comprehensive clinical characterization of the early stages of DLB, focusing on motor and non-motor symptoms. Our main findings highlight that motor symptoms in early-stage DLB are as frequent as in PD, a key feature that distinguishes DLB from AD. Also, non-motor symptoms are highly prevalent in DLB, even more so than in PD, and notably more frequent than in AD, emphasizing their importance in distinguishing between these conditions. Motor and non-motor symptoms collectively contribute to a greater impact on daily functioning, reduced functional independence, and poorer quality of life in DLB patients, compared to PD and AD patients.

Although parkinsonism is one of the main diagnostic core features for DLB, the presence and phenomenology of motor symptoms in DLB exhibit considerable variability across studies. The presence of parkinsonism varies from 15 to 70% in DLB patients at early stages (10–14). In the largest study with pathologically confirmed prodromal DLB patients, 68.5% of the 111 cases showed parkinsonian signs (14). However, the disease duration was not specified in this report. Other studies focusing on the prodromal stages of DLB found that parkinsonism is the most common core symptom during the prodromal phase of DLB (13) and that motor changes, such as slowness and parkinsonian gait, can occur up to 5 years before DLB diagnosis (33). Our study, focusing on symptoms of DLB beyond cognitive decline, specifically in motor features, found that all DLB patients showed parkinsonism within the first 2 months after diagnosis. This feature is of great importance, since could help in distinguishing DLB from AD in the clinical yard. Additionally, after classifying DLB patients using the motor subgroups commonly defined in PD, we found that 65.2% of DLB patients presented with the PIGD phenotype. The prevalence of tremor in DLB patients varies widely in the literature, from 10 to 55.4%, with most studies only noting its presence (6, 14, 33–35). In our cohort, 73.9% of DLB patients reported tremor, which was predominantly unilateral and more frequent on the right side, with action tremor being more common than rest tremor. We found no significant differences between DLB and PD patients in the presence of tremor, bradykinesia, hypophonia or micrographia; however, falls and gait disturbances were more frequent in DLB, and the PIGD phenotype was more prevalent in DLB compared to PD. This greater prevalence of falls and gait disturbances in DLB has also been reported in other studies comparing DLB to PD (36, 37). Given the clinical similarity between parkinsonism in DLB and PD, it is crucial to assess for DLB symptoms to anticipate potential side effects of dopaminergic treatment and, if needed, consider antipsychotics or acetylcholinesterase inhibitors (AChEIs). AD patients in our cohort exhibited overall fewer motor symptoms, consistent with previous studies (6, 13, 14, 34). The variability observed across studies in the presence of motor symptoms in DLB patients may be attributed to differences in study designs, patient populations, the stage of the disease at the time of evaluation, and the fact that many studies originate from memory clinics, where motor symptoms might not be systemically assessed.

Regarding non-motor symptoms, our findings align with previous literature. We observed a similar prevalence of RBD as in other studies that have clinically diagnosed RBD (10, 12, 14, 36). Additionally, DLB patients exhibited similar autonomic dysfunction, with greater involvement in urinary, gastrointestinal and sexual domains, similar to PD patients. However, they showed more abnormalities in orthostatic test compared to PD and AD patients. Other studies have reported more frequent autonomic symptoms in DLB (11, 13, 15, 36). Neuropsychiatric symptoms, such as major visual hallucinations, were more frequent in DLB, leading to a greater impact on quality of life and higher caregiver distress compared to PD and AD, which has also been previously reported in the literature (13, 14, 34–36). Interestingly, in the previously mentioned large study of 111 pathologically confirmed prodromal DLB, it was found that a history of visual hallucinations increased the risk of DLB diagnosis by almost 12-fold compared to AD (*p* < 0.0001), although only 10% of DLB patients showed this symptom in the prodromal stage (14). Our findings regarding non-motor symptoms others than cognitive decline, in line with other reported in the literature, highlights the importance of the anamnesis and physical examination focused on non-motor symptoms, which is essential for the differential diagnosis of DLB with other pathologies that cause cognitive decline, even in the most initial phases of the disease.

For many years, there has been considerable debate about whether DLB and PD are expressions of the same underlying disease (38–40). The term Lewy body disease (LBD) encompasses PD, PD dementia (PDD) and DLB. Indeed, PDD and DLB are clinically very similar, particularly in terms of cognitive, psychiatric, and RBD symptoms (41–44), with the primary difference being the timing of dementia and parkinsonism onset, a distinction referred to as the “one-year rule,” which is considered purely arbitrary by some researchers (45, 46). Neurobiologically, DLB and PDD also share significant similarities, with overlapping *in vivo* biomarkers and common neuropathological features, such as widespread cortical Lewy body deposition (45, 47, 48). However, neuropathological differences between PDD and DLB have been identified. Studies indicate that DLB is associated with higher comorbid AD pathology, more severe cortical Lewy body and tau pathology, and a greater frequency of cerebral amyloid angiopathy compared to PDD (49–51). AD pathology likely influences the clinical presentation of DLB, leading to an accelerated disease course, poorer cognitive outcomes, and worse global prognosis (52). Despite the significant impact of AD copathology, it is not always routinely assessed in patients with LBD. In our study, newly diagnosed DLB and PD patients exhibited similar motor symptoms with no significant differences in MDS UPDRS III scores, the percentage of patients treated with levodopa, or their LEDD. This finding further supports the theory that DLB and PD may represent a continuum of the same disease. Furthermore, the fact that motor symptoms in this cohort of early DLB patients are as prominent as those in PD, lead us to remark the importance of identifying and treating motor symptoms in DLB patients.

The main strengths of this study are the focus in early DLB patients, the inclusion of selected comparison groups with PD and AD individuals, as well as controls without neurologic diseases, and the comprehensive assessment of motor and nonmotor symptoms. However, several limitations should be considered when interpreting the results. Firstly, the small sample size and the unicentric design may limit the spread of our findings. Secondly, it is possible that we are missing a proportion of probable DLB patients without parkinsonism, as previous literature has described that approximately 15% of DLB patients have negative DaT scans and clinically present to none-to-mild motor parkinsonism (53). Thirdly, RBD was determined clinically without confirmation via polysomnography, which could have led to under- or over-diagnosis of RBD. Also, study groups were not matched based on gender or age. Additionally, diagnostic delay was defined as the time between motor or cognitive symptom onset and diagnosis, without exact dates for sleep or psychiatric symptoms. Furthermore, the lack of control for the use of AChEIs when comparing DLB and AD groups could present a potential source of bias. However, this was not controlled in our study because most patients who were receiving AChEIs had only recently started treatment, with a median time from diagnosis to the study visit of only 2 months. Lastly, the lack of neuropathological confirmation of the diagnosis leaves room for potential misdiagnosis in some patients. Further studies are warranted to explore and confirm the motor symptom profile in patients with prodromal DLB, in addition to longitudinal studies with CSF and blood biomarkers and neuropathological confirmation.

In conclusion, our findings indicate that motor symptomatology in recently diagnosed DLB patients significantly overlaps with that observed in PD. Also, the greater presence of non-motor symptoms, such as autonomic dysfunction and neuropsychiatric manifestations, in DLB patients compared to AD and PD, further adds complexity to the clinical presentation of these patients. Both, motor and non-motor symptoms should be considered in the differential diagnosis of DLB and other diseases causing cognitive decline, such as AD. Future longitudinal studies, incorporating biomarker analyses and neuropathological confirmation, are essential to clarify the complexities of this disease, improve diagnostic accuracy, and develop targeted therapeutic interventions.

## Data Availability

The raw data supporting the conclusions of this article will be made available by the authors without undue reservation.
